# Au_101_–rGO nanocomposite: immobilization of phosphine-protected gold nanoclusters on reduced graphene oxide without aggregation†

**DOI:** 10.1039/d0na00927j

**Published:** 2021-01-07

**Authors:** Hanieh Mousavi, Yanting Yin, Liam Howard-Fabretto, Shailendra Kumar Sharma, Vladimir Golovko, Gunther G. Andersson, Cameron J. Shearer, Gregory F. Metha

**Affiliations:** Department of Chemistry, University of Adelaide Adelaide SA 5005 Australia cameron.shearer@adelaide.edu.au greg.metha@adelaide.edu.au; Flinders Centre for NanoScale Science and Technology, Flinders University Adelaide SA 5001 Australia; The MacDiarmid Institute for Advanced Materials and Nanotechnology, School of Physical and Chemical Sciences, University of Canterbury Christchurch 8140 New Zealand

## Abstract

Graphene supported transition metal clusters are of great interest for potential applications, such as catalysis, due to their unique properties. In this work, a simple approach to deposit Au_101_(PPh_3_)_21_Cl_5_ (Au_101_NC) on reduced graphene oxide (rGO) *via* an *ex situ* method is presented. Reduction of graphene oxide at native pH (pH ≈ 2) to rGO was performed under aqueous hydrothermal conditions. Decoration of rGO sheets with controlled content of 5 wt% Au was accomplished using only pre-synthesised Au_101_NC and rGO as precursors and methanol as solvent. High resolution scanning transmission electron microscopy indicated that the cluster size did not change upon deposition with an average diameter of 1.4 ± 0.4 nm. It was determined that the rGO reduction method was crucial to avoid agglomeration, with rGO reduced at pH ≈ 11 resulting in agglomeration. X-ray photoelectron spectroscopy was used to confirm the deposition of Au_101_NCs and show the presence of triphenyl phosphine ligands, which together with attenuated total reflectance Fourier transform infrared spectroscopy, advocates that the deposition of Au_101_NCs onto the surface of rGO was facilitated *via* non-covalent interactions with the phenyl groups of the ligands. Inductively coupled plasma mass spectrometry and thermogravimetric analysis were used to determine the gold loading and both agree with a gold loading of *ca*. 4.8–5 wt%. The presented simple and mild strategy demonstrates that good compatibility between size-specific phosphine protected gold clusters and rGO can prevent aggregation of the metal clusters. This work contributes towards producing an agglomeration-free synthesis of size-specific ligated gold clusters on rGO that could have wide range of applications.

## Abstract 2

A method is presented for the deposition of gold clusters onto reduced graphene oxide, without aggregation of the gold clusters.

## Graphical abstract

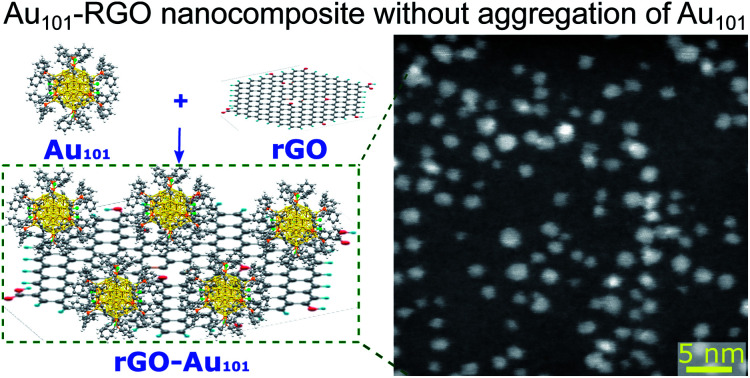

## 1 Introduction

In recent decades, gold clusters (AuNCs) with sizes of less than 2 nm have been attracting increased attention due to their unique properties compared to larger gold nanoparticles and bulk gold.^1,2^ Size-specific clusters can offer high atomic efficiency due to high surface to-volume ratio with most atoms being at or close to the surface. Also, the strongly size-dependent electronic properties of ultra-small metal clusters offer the opportunity to tune their reactivity by choice of appropriate size and composition.^3,4^ Furthermore, such clusters are small enough to perform high level DFT calculations,^5–7^ which are crucial for comprehensive understanding of the catalytic mechanisms needed for the development of superior catalysts.^8^ Therefore, size-specific clusters are considered as a platform for investigation of catalysts at the atomic level.^9^ However, AuNCs need to be protected and stabilized with ligands during their synthesis to stop uncontrolled particle growth and aggregation.^10^ Triphenylphosphine (PPh_3_) ligands are often employed in the synthesis of phosphine-protected gold clusters as this approach allows fabrication of ultra-small clusters. Importantly, good control over the cluster metal core size can allow narrow particle size distributions (as in the case of Au_101_(PPh_3_)_21_Cl_5_), while many other clusters are synthesised with atomic precision (*i.e.* no size distribution with all species having exact same number of metal atoms as in the case of Au_9_(PPh_3_)_8_(NO_3_)_3_). In addition, the electronic structure and reactivity of the clusters could be improved due to electronic and steric factors imposed by the phosphine ligands.^11^ As a result, phosphine-protected gold clusters formulated as Au_*n*_(PPh_3_)_*m*_, such as Au_9_(PPh_3_)_8_(NO_3_)_3_ and Au_101_(PPh_3_)_21_Cl_5_, have been shown to exhibit catalytic activity in a wide range of reactions, such as hydrogenation of terminal alkynes into alkenes,^12^ CO oxidation,^13,14^ styrene oxidation to benzaldehyde^15^ and styrene epoxide,^16^ and benzyl alcohol oxidation.^17,18^ However, the size-specific catalytic activity of AuNCs has often been difficult to determine experimentally as clusters are prone to aggregation either during deposition or activation on the support,^19,20^ or deactivation of the catalyst following initial reaction.^21^ Therefore, methods for the fabrication of gold cluster-based catalysts resilient to aggregation are in high demand in nanomaterials,^22^ catalysis^4,16,23,24^ and sensing communities.^25,26^

It has been shown that the successful deposition of AuNCs on surfaces depends on various factors, such as the type of support and ligand, synthetic conditions and cluster–support interactions.^9^ Therefore, understanding these factors is crucial in the design of deposition method.

It has been demonstrated that small metal nanoparticles (not size specific) such as Ag,^27^ Pd^28^ and Au,^21,29,30^ can be immobilized on reduced graphene oxide (rGO).^31^ rGO has a two-dimensional structure with extraordinary properties, such as high electrical conductivity, large surface area and high charge mobility.^32^ Moreover, rGO has the potential to be involved in non-covalent interactions with clusters through π–π stacking with the protecting organic ligands, such as PPh_3_.^33^

There are a few recent examples of nanocomposites containing small gold nanoparticles (not size specific) and rGO supports, yet most of the previously used synthesis methods involve the use of harsh reagents, such as ammonia^34,35^ and hydrazine hydrate.^30^ For these examples, the gold nanoparticles are formed *in situ* during nanocomposite synthesis. Furthermore, to the best of our knowledge, research on size-specific triphenylphosphine-protected AuNCs supported on rGO has not been reported.

Herein, we present a simple and rapid method to produce a nanocomposite based on pre-synthesised AuNCs and rGO without any additional pre-treatment of this support *via* an *ex situ* method at room temperature (RT). The reduction of GO occurs in the absence of ammonia in aqueous media; rGO is consequently washed and suspended in methanol. The PPh_3_ ligands act as tethers creating a strong interaction with rGO and enabling uniform distribution of Au_101_NCs on the surface of rGO without any aggregation even at the relatively high metal loading used here. The properties and formation mechanism of the Au_101_NCs–rGO nanocomposite are discussed.

## 2 Experimental section

### 2.1 Reagents and materials

All chemicals used as received throughout the study, unless otherwise stated: natural graphite flakes (Uley, Eyre Peninsula, South Australia), 98% sulfuric acid (H_2_SO_4_, RCI Labscan), 85% phosphoric acid (H_3_PO_4_, Chem-Supply), 70% nitric acid (HNO_3_, Chem-Supply), 32% hydrochloric acid (HCl, RCI Labscan), 30% hydrogen peroxide (H_2_O_2_, Chem-Supply), potassium permanganate (KMNO_4_, Merck), methanol (CH_3_OH, Merk, Analysis Grade), high-purity Milli-Q water (18.2 MΩ cm at 25 °C), 99% triphenylphosphine (PPh_3_, Merck), and gold single component standard ICP (TraceCERT, Merck, 999 mg L^−1^), 68 Component ICP-MS Standard (High Purity Standards, HPS, 10 μg mL^−1^).

### 2.2 Preparation of graphene oxide (GO)

GO was prepared using the improved Hummers' method.^36^ Natural graphite flakes were ground and sieved to a particle size < 150 μm. A mixture of concentrated H_2_SO_4_ and H_3_PO_4_ with 9 : 1 ratio (by volume) was cooled down to 4 °C and then slowly poured into a mixture of graphite powder (3.0 g) and KMnO_4_ (18.0 g). The mixture was heated up to 50 °C and magnetically stirred for 12 h, followed by cooling down to room temperature. The mixture was poured onto ice (∼200 mL) in the presence of 30% H_2_O_2_ (3 mL). Subsequently, the resulting material was magnetically stirred for 2 h to ensure sufficient exfoliation to GO. The product was then washed multiple times with MilliQ water until pH ∼ 2 and centrifuged at 4000 rpm for 30 min (Sigma 2-16 P) to recover GO solid product during each wash before re-dispersing it in the fresh portion of MilliQ by sonication (Elmasonic P).

### 2.3 Preparation of reduced graphene oxide (rGO)

Reduction of GO was carried out using the hydrothermal method at 190 °C for 12 h in a 500 mL Teflon-lined autoclave.^37^ The reduction suspensions were prepared using GO aqueous solution (8.3 mg mL^−1^) in MilliQ water. Due to the acidic process of graphite oxidation, this solution had a low pH ≈ 2 after diluting with water. The resultant black product was then collected by centrifugation at 4000 rpm and the supernatant liquid decanted away. The remaining material was first washed multiple times with MilliQ water (as described in 2.2 above) until neutral pH (Mettler-Toledo FG2 pH meter) of supernatant liquid was achieved. The obtained product was then washed three times with methanol in a similar manner. In order to obtain a stable suspension, the rGO was suspended in methanol (7.32 mg mL^−1^) *via* sonication in an ice-bath using probe sonication (Branson 450 Digital Sonifier, 400 W) for 10 minutes at 60% power to obtain a well-dispersed rGO suspension. Obtained suspensions were kept in the fridge and did not show any sign of flocculation for over a year. For comparison experiments, the pH of GO was adjusted employing ammonia to 11 and the resulting solution was hydrothermally treated and processed in the same manner described above.

### 2.4 Preparation of Au_101_NC–rGO nanocomposite

**Fig. 1:**
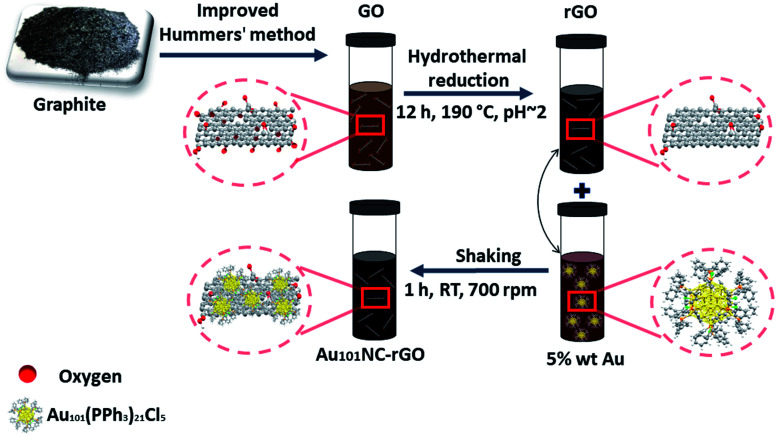
Schematic of synthesis of Au_101_NC–rGO showing oxidation of graphite to GO, hydrothermal reduction in acidic aqueous media to create rGO and mixing with Au_101_NC in methanol to form Au_101_NC–rGO.

The procedure for fabrication of rGO sheets with controlled 5 wt% Au loading of Au_101_NCs *via ex situ* method is schematically presented in Fig. 1.

Au_101_(PPh_3_)_21_Cl_5_ was synthesized as reported by Hutchison and co-workers.^38^ 5 mg of Au_101_(PPh_3_)_21_Cl_5_ was dispersed in 5 mL of methanol *via* bath sonication (Elmasonic P) for 10 min at RT to obtain a homogeneous dispersion. The obtained suspension was then wrapped immediately with aluminium foil to minimize the effect of light on the Au_101_NC and was kept in the fridge.

Typically, to make 5 mg Au_101_NC–rGO nanocomposite with 5 wt% Au loading in 1.5 mL methanol, 0.32 mL of the as-obtained Au_101_NC dispersion (which corresponds to 0.25 mg non-ligated or ∼0.32 mg ligated Au_101_) was added slowly dropwise to the magnetically stirred as-synthesized rGO dispersion (4.68 mg in 0.64 mL) at RT and made up to 1.5 mL with methanol. Then, it was wrapped immediately with aluminium foil to minimize the effect of light, followed by mixing using an orbital shaker (THERMOstar) for 1 h at RT at 700 rpm. Obtained product was kept in the fridge; the dispersion did not show signs of flocculation over six months.

### 2.5 Preparation of PPh_3_–rGO composite (control samples)

The procedure to make 5 mg PPh_3_–rGO composite in 1.5 mL methanol, containing the same quantity of PPh_3_ as the 5 wt% Au nanocomposites, is similar to the preparation of Au_101_NC–rGO nanocomposite described in Section 2.4. The only difference is that a solution of PPh_3_ (2.7 mg) in methanol (5 mL) was made. An aliquot (0.13 mL) of as-obtained PPh_3_ solution containing ∼0.07 mg PPh_3_ was added slowly dropwise to the as-synthesized rGO dispersion (4.93 mg in 0.673 mL) at RT and made up to 1.5 mL with methanol.

### 2.6 Characterization

rGO, Au_101_NC and Au_101_NC–rGO nanocomposite were analysed by different characterization techniques including attenuated total reflectance Fourier transform infrared spectroscopy (ATR-FTIR), transmission electron microscopy (TEM), scanning electron microscopy (SEM), high-angle annular dark-field scanning transmission electron microscopy (HAADF-STEM), inductively coupled plasma mass spectrometry (ICP-MS), X-ray photoelectron spectroscopy (XPS), ultraviolet-visible absorption spectroscopy (UV-Vis) and thermogravimetric analysis (TGA). Detailed descriptions of the characterization protocols are given in the ESI.†

## 3 Results and discussion

### 3.1 Characterization of rGO

**Fig. 2:**
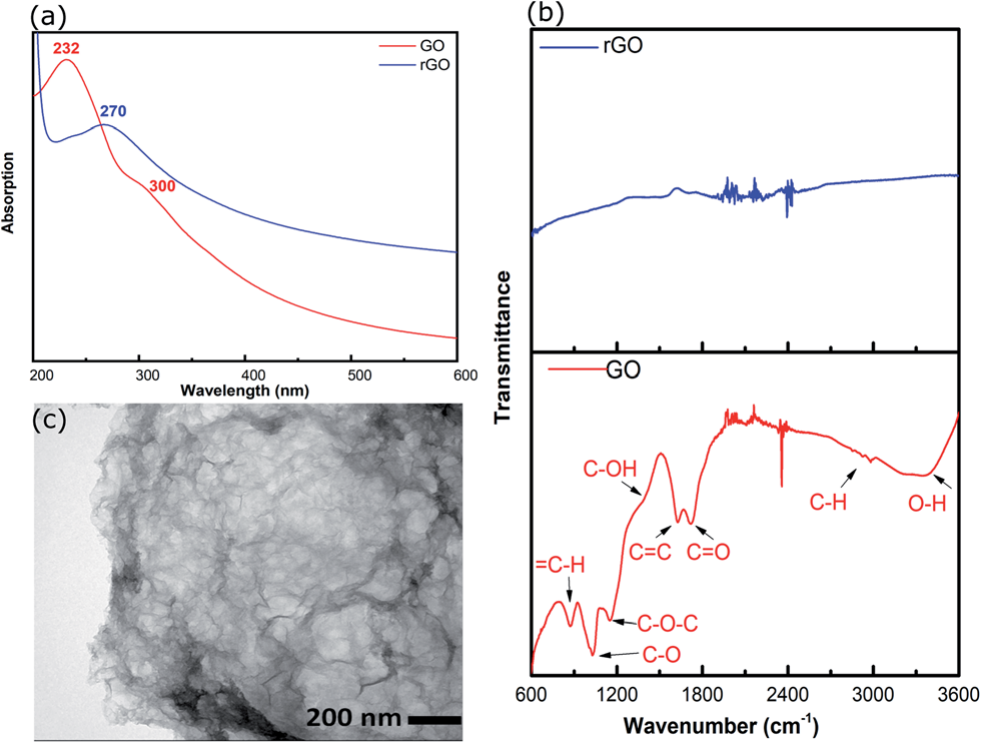
(a) UV-vis spectra of GO and rGO, (b) ATR-FTIR spectra of GO and rGO, and (c) TEM image of rGO.

rGO was prepared by hydrothermal reduction in aqueous solution (pH ∼2) and redispersed in methanol. Methanol was chosen as the solvent because it is compatible with a range of AuNCs including Au_101_NCs. Characterisation was completed to confirm reduction of GO and morphology of rGO flakes. GO and rGO display a set of distinctive spectroscopic characteristics which distinguish them. UV-vis absorption spectroscopy was employed to confirm the reduction of GO; the spectra of GO and rGO are shown in Fig. 2(a). The spectrum of GO shows a strong maximum at 232 nm, which can be assigned to C=C–C π–π* transitions, and a weak shoulder at 300 nm due to C=O n–π* transitions.^39^ After the reduction of GO, the strong absorption peak at 232 nm shifts to 270 nm due to the restoration of the sp^2^ network, as commonly observed during GO reduction.^40^

ATR-FTIR was applied to identify functional groups on GO and rGO and determine the degree of oxidation/reduction; the spectra of GO and rGO are presented in Fig. 2(b). The GO exhibited several characteristic absorption bands due to oxygen-containing functional groups. These peaks decreased dramatically in intensity, or even disappeared, due to the reduction of GO, which is consistent with previous reports.^36,41,42^

The morphology of rGO was investigated using TEM. The TEM image of rGO in Fig. 2(c) displays an ultrathin sheet with folds and scrolls at the edges and a variety of ripples and wrinkles on the surface.

### 3.2 Characterization of Au_101_NC

As will be seen later, it is important to fully characterise the gold cluster, including the PPh_3_ ligands. The ATR-FTIR spectra of Au_101_NC and PPh_3_ are shown in Fig. S3.† The absorption bands at 690, 740, 1000–1100, 1430 and 1475 cm^−1^ (pink boxes) contain the signatures of PPh_3_. The bands at 690, 1100, 1430, and 1475 cm^−1^ correspond to the C–C asymmetric stretch of phenyl groups attached directly to P (P–C_(Ph)_), and the peak at 740 cm^−1^ can be assigned to the CH_(Ph)_ (out-of-plane) vibration.^45^ Other bands revealed at 1000, 1020 and 1070 cm^−1^ are attributed to ring breathing of C=C_(Ph)_ and CH_(Ph)_ (in-plane) vibrations respectively.^46^ Overall, the spectra indicate the presence of PPh_3_ ligands attached to the Au_101_ core.

The TGA results (in N_2_) for the Au_101_NC and PPh_3_ are given in Fig. S4.† It is seen that PPh_3_ weight loss occurs between 110–245 °C, while the complete removal of PPh_3_ ligands from the Au_101_NC core occurs between 150 °C and 350 °C. This shift to higher temperature indicates that PPh_3_ is strongly attached to the gold cluster core. For Au_101_NC, the weight loss showed a percentage loss of 24.1%, which is close to the theoretical amount of 22.2% (loss of PPh_3_ and Cl), based on the chemical formula Au_101_(PPh_3_)_21_Cl_5_.^38^

**Fig. 3:**
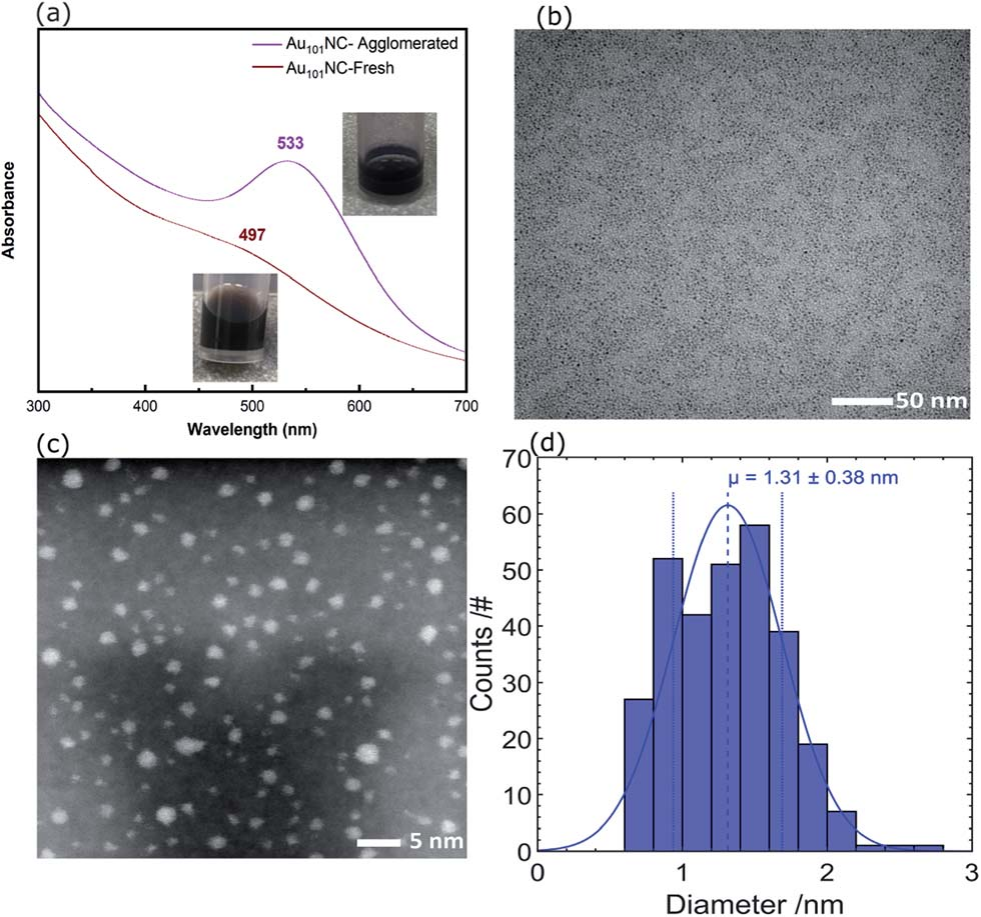
(a) UV-vis spectra of agglomerated and fresh Au_101_NC in methanol, (b) TEM image, (c) HAADF-STEM, and (d) size distribution histogram of Au_101_NC dropcast onto TEM grid from methanol solution.

The UV-vis absorption spectrum of Au_101_NC in methanol is shown in Fig. 3(a) (lower trace). It is clearly seen that there is no localised surface plasmon resonance (LSPR) absorption, indicating that the clusters are non-metallic and retain the small size of Au_101_NC, less than 2 nm.^18^ To compare with an agglomerated form of Au_101_NC, the dispersion was left under ambient conditions (including light) for 2 weeks and the UV-vis absorption was re-measured. As seen in the upper trace of Fig. 3(a), the appearance of a LSPR band at 533 nm indicates the formation of gold nanoparticles greater than 2 nm.^18^

For further investigation, the size distribution of Au_101_NC dispersed in methanol was analysed by TEM; a representative image is shown in Fig. 3(b). The image shows a homogenous and narrow distribution of Au_101_NC. Additionally, high resolution HAADF-STEM image and size-distribution histogram, shown in Fig. 3(c) and (d), respectively, indicate an average diameter of 1.3 ± 0.4 nm. It is important to note that 26% of the clusters have diameters smaller than 1 nm and 3% larger than 2 nm, in agreement with previously reported values (1.5 ± 0.4 nm).^38^ This will be further addressed when discussing the XPS results (*vide infra*).

### 3.3 Characterization of Au_101_NC–rGO nanocomposite

The composite of Au_101_NC–rGO was prepared by gently mixing methanol suspensions of rGO and Au_101_NC in the dark at room temperature. This mild procedure was chosen to reduce the likelihood of AuNCs agglomerating and forming nanoparticles (>2 nm) during deposition. Characterization of the nanocomposite, Au_101_NC–rGO, was performed to determine structural features, morphology, composition, degree of agglomeration, and to provide an evaluation of the interactions between the AuNCs and rGO.

**Fig. 4:**
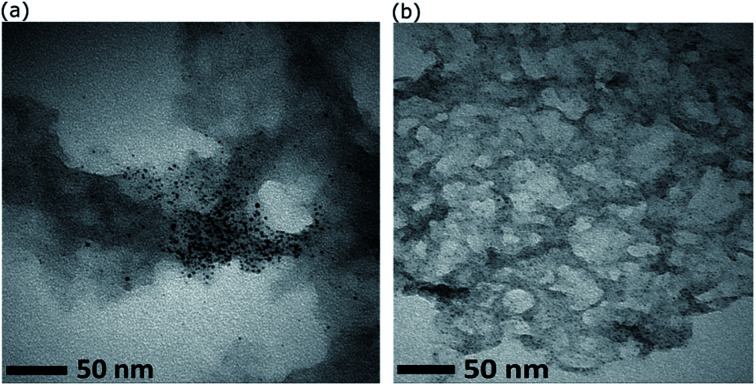
TEM images showing the effect of pH on size and distribution of Au_101_NC on rGO reduced hydrothermally at (a) pH ∼ 11, and (b) pH ∼ 2.

Initial experiments were conducted using AuNCs deposited onto rGO produced by hydrothermal reduction subjected to ammonia (pH ∼11). This is the most common hydrothermal reduction method for GO.^34,35^ As can be seen in Fig. 4(a), this results in significant non-homogeneity and agglomeration of AuNCs. The gold particle size histogram (Fig. S5†) was found to have a log-normal distribution with mode of 2.57 nm (mean = 3.5 ± 2.6). Therefore, an investigation of the influence of pH (used during reduction of GO) on the gold particle size upon deposition of AuNCs was conducted. It was observed that Au_101_NCs are sensitive to aggregation at basic pH with Au_101_NC solutions turning blue (indicating agglomeration) upon addition of small amounts of dilute NaOH to Au_101_NC dispersion in water. In comparison, when adding a small amount of HCl to Au_101_NC in water we observed no colour change. We also determined that Au_101_NC solutions have a natural acidic p*K*_a_ from dispersion in water. Consequently, the TEM image in Fig. 4(b) shows that when GO is reduced at low pH (∼2), deposition of Au_101_NC results in material with ultra-small gold particles with narrow particle size distribution decorating the surface of rGO. This simple change to the GO reduction procedure clearly results in a marked improvement in nanocomposite preparation. A full spectroscopic comparison of rGO (UV-Vis, FTIR, Raman, XPS) is shown in the ESI,† with the major difference being the presence of nitrogen in the rGO reduced in basic conditions. We believe the basic surface groups on rGO are causing the agglomeration of gold. All subsequent experiments focus on rGO produced at low pH.

**Fig. 5:**
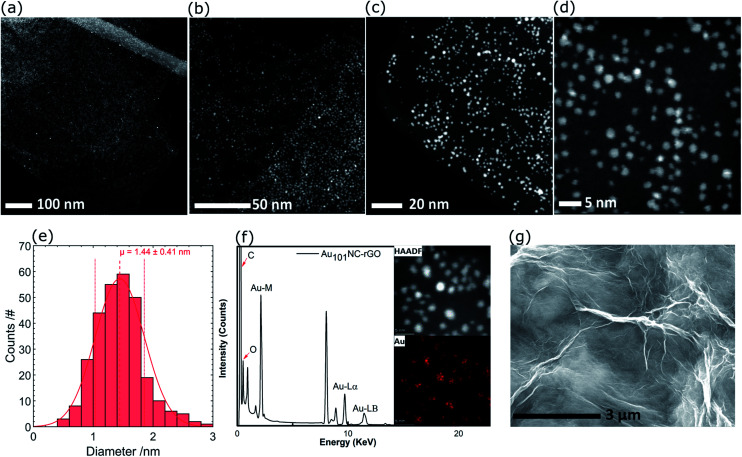
(a–d) HAADF-STEM images of Au_101_NC–rGO with different magnifications, (e) Au particle size distribution histogram, (f) HAADF and elemental mapping of Au, and (g) SEM image of Au_101_NC–rGO.

Representative high resolution HAADF-STEM images for the as-prepared Au_101_NC–rGO nanocomposite (with rGO made at low pH) are presented in Fig. 5(a–d) and the size-distribution histogram is shown in Fig. 5(e). The Au_101_NCs have average diameter of 1.4 ± 0.4 nm, including about 8% of gold particles with diameter greater than 2 nm, indicating that the deposition procedure has not significantly changed the cluster size (compare to Fig. 3(c and d)). In comparison, earlier reports on Au_101_NCs deposited on supports such as TiO_2_,^18^ SiO_2_,^18^ WO_3_,^47^ and activated carbon^48^ mention gold particle sizes of 2.0–2.7, ∼3.6, ∼2.2, and ∼2.6 nm, respectively, indicating agglomeration of Au_101_NCs on these supports. In addition, HAADF-STEM elemental mapping images obtained using energy-dispersive X-ray spectroscopy (EDX) collected at each pixel in the image (Fig. 5(f)) confirms that the ∼1.4 nm sized features observed are composed of gold. STEM-EDX elemental mapping for P is more difficult to determine since the P K_α_ peak overlaps with the more intense Au M_α_ line. Nevertheless, it is possible to show that P is also co-located on the cluster cores, as shown in the STEM-EDX map and spectrum in Fig. S6.†

The SEM of Au_101_NC–rGO nanocomposite shown in Fig. 5(g) is very similar to that seen for rGO (Fig. S7†). The similarity of the support morphology indicates that the Au_101_NC has integrated uniformly with the rGO to form the Au_101_NC–rGO nanocomposite without affecting the morphology of the rGO support.

ICP-MS of Au and P was used to determine the loading of AuNCs onto rGO. Instead of completing acid digestion of the Au_101_NC–rGO nanocomposite (with concomitant matrix dissolution problems), the residual solvent (*i.e.* after forming the nanocomposite) was analysed, which is a commonly used method.^49^ This yielded the amount of Au and P not adsorbed to rGO, which is then used to calculate a wt% loading. 99.3 ± 0.5% of Au was adsorbed while 85.2 ± 4.0% of P was adsorbed. This equates to an Au wt% loading of 4.9 wt% (Table 1).

**Table 1:** % adsorption and weight loading (wt%) of Au and P on rGO determined by ICP-MS and TGA

Characterization	% Au adsorbed	% P adsorbed	Au loading (wt%)
ICP-MS	99.3 ± 0.5	85.2 ± 4.0	4.9 ± 0.5
TGA	94.0 ± 0.1	—	4.7 ± 0.1

Interestingly, the higher residual% P than % Au in the solvent after Au_101_NCs deposition, corresponds to approximately 4 extra PPh_3_ ligands remaining in solution per Au cluster, indicating that there are ∼17 PPh_3_ ligands which could be still attached to each Au_101_NC on rGO (P : Au of 0.17).

To evaluate the interaction of Au_101_NC with the rGO surface, a combined study using XPS, ATR-FTIR, TGA, and UV-vis was employed.

**Fig. 6:**
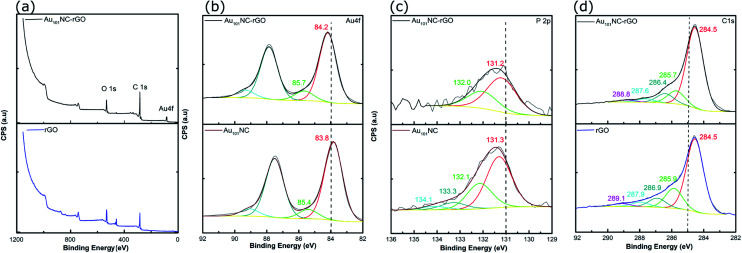
(a) Overview of XPS spectra of rGO and Au_101_NC–rGO (b) Au 4f spectra of Au_101_NC and Au_101_NC–rGO, (c) P 2p spectra of Au_101_NC and Au_101_NC–rGO, and (d) C 1s spectra of rGO and Au_101_NC–rGO.

Samples of Au_101_NC, rGO and Au_101_NC–rGO nanocomposite were investigated by XPS to determine the elemental composition and chemical environment. Based on our previous XPS studies of Au clusters on various supports,^19^ the degree of agglomeration of Au_101_NCs to bulk-like large nanoparticles (*i.e.* >2 nm) can also be estimated. The XPS survey spectrum of rGO (Fig. 6(a)-lower trace) revealed two main peaks, C 1s and O 1s. The XPS survey spectrum of the Au_101_NC–rGO nanocomposite, Fig. 6(a)-upper trace, shows peaks due to Au 4f as well as C 1s and O 1s.

Within the Au 4f region of Au_101_NC in Fig. 6(b)-lower trace, there are two sets of doublets, due to the 4f_7/2_ and 4f_5/2_ spin–orbit components that are separated by 3.7 eV. The Au 4f_7/2_ component shows a major peak at 84.2 ± 0.54 eV, and a minor peak at 85.7 eV ± 0.58 eV. We have previously referred to these peaks as the low binding peak (LBP) and high binding peak (HBP), respectively; the former is specifically due to Au_101_NC and the latter is due to smaller clusters perhaps even Au_1_ (a by-product of the cluster synthesis).^19^ The presence of smaller clusters is also seen in the images and histogram from the HAADF-STEM, shown in Fig. 3(d). It is not clear if these smaller clusters are present in the prepared Au_101_NC or whether they occur upon dissolution in the methanol. The Au 4f region of the Au_101_NC–rGO nanocomposite, Fig. 6(b)-upper trace, has a similar set of features to that observed for pure, unsupported Au_101_NCs; interestingly both peaks show a ∼0.4 eV shift towards higher binding energy upon deposition on rGO. It is also evident that the smaller Au clusters are present in the nanocomposite in approximately the same % of the total gold population (11.1% and 12.9%, respectively, see Table S1†).

Analysis of the P 2p region for the pure, unsupported cluster, Au_101_NC, (Fig. 6(c)-lower trace), shows a broad peak which was fit to two sets of doublets, due to the 2p_3/2_ and 2p_1/2_ spin–orbit components that are separated by 0.87 eV. The P 2p_3/2_ component shows a major peak at 131.3 ± 0.55 eV, due to triphenylphosphine bound to the gold cluster core, and a minor peak at 133.3 eV, which is possibly a trace amount of triphenylphosphine oxide. The P 2p region of the Au_101_NC–rGO nanocomposite (upper trace) shows only a single doublet with P 2p_3/2_ at 131.2 ± 0.64 eV, indicating that the triphenylphosphine ligands remain attached to the Au cluster after deposition onto rGO.^38,50^ The P : Au atomic ratio is 0.26 ± 0.04 for Au_101_NC and 0.25 ± 0.04 for Au_101_NC–rGO, further indicating that the number of ligands remaining attached to the gold cluster gold is relatively unchanged upon deposition onto rGO (theoretical value based on the formulae Au_101_(PPh_3_)_21_Cl_5_ is 0.21). This is similar to the ICPMS results (*vide supra*), which showed a P : Au ratio of 0.17.

The C 1s region of rGO and Au_101_NC–rGO nanocomposite can be used to determine the chemical nature of carbon in the system. The spectrum of rGO, Fig. 6(d)-lower trace, shows five peaks centred at 284.5, 285.9, 286.9, 287.9 and 289.1 eV, which can be assigned to C=C–C (sp^2^), C–C (sp^3^), C–OH, C–O–C/C=O and O–C=O, respectively.^43,44^ In the Au_101_NC–rGO nanocomposite (upper trace), the same five components are observed, indicating that the relative abundance of the different carbon groups in rGO and Au_101_NC–rGO are very similar. The fraction of each functional group present in rGO, Au_101_NC, and Au_101_NC–rGO nanocomposite, and the analysis from deconvolution of C 1s, O 1s, Au 4f and P 2p spectra are shown in Tables S1 and S2.†

**Fig. 7:**
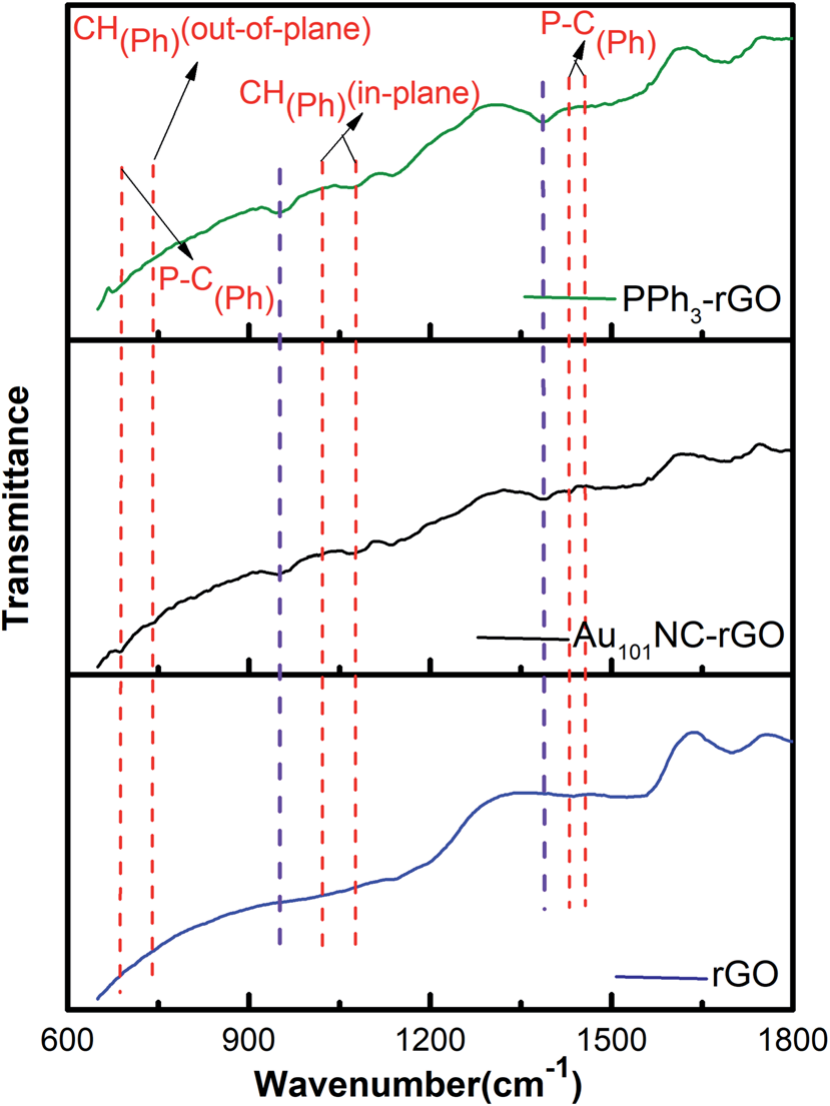
The ATR-FTIR spectra of rGO, Au_101_NC–rGO, and PPh_3_–rGO.The red dashed vertical lines indicate features associated with PPh_3_, the purple dashed vertical lines are associated with new features observed in the nanocomposite but not in rGO or PPh_3_.

ATR-FTIR study was also undertaken to monitor any functional group changes upon interaction of Au_101_NCs with the rGO surface. The ATR-FTIR spectra of rGO, Au_101_NC–rGO, and PPh_3_–rGO are presented in Fig. 7(a). The red dashed lines indicate features arising from the PPh_3_ ligands. The major PPh_3_ features remain in the Au_101_NC–rGO although they have diminished in intensity compared to the spectra of pure PPh_3_ and Au_101_NC (Fig. S3†) due to the low ligand content of the composite (<1%). The observation of phenyl peaks confirms the presence of ligands from Au_101_NC on the surface of rGO, which supports assignment to PPh_3_ of the P 2p peak observed in XP spectrum. Furthermore, the Au_101_NC–rGO spectrum reveals new, albeit weak, peaks at ∼1390 and ∼950 cm^−1^ (purple dashed lines). These new absorption peaks are difficult to assign with confidence, but the peak at 950 cm^−1^ is in the general region for C–O–C(aryl) and P–O–C(aryl).^51^ They are not observed in Au_101_NC nor rGO but are seen in the spectrum of PPh_3_–rGO and therefore potentially arise from an interaction of the PPh_3_ ligands with rGO.

**Fig. 8:**
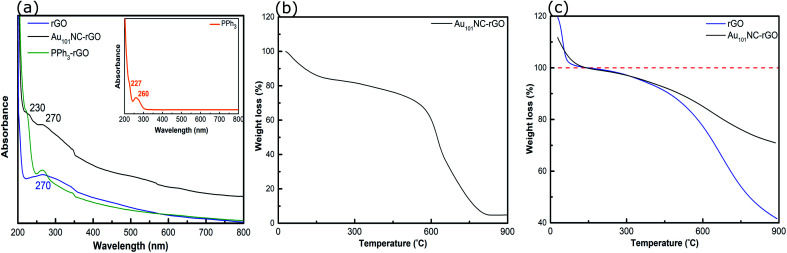
(a) UV-vis spectra of rGO, PPh_3_–rGO and Au_101_NC–rGO, (b) TGA curve of Au_101_NC–rGO (in air), and (c) TGA curves of rGO and Au_101_NC–rGO (in N_2_). Insert in (a) shows the UV-vis spectrum of PPh_3_.

The UV-vis spectra of PPh_3_, PPh_3_–rGO and Au_101_NC–rGO are presented in Fig. 8(a). The spectrum of PPh_3_ (inset) exhibits two peaks at 227 and 260 nm, which are assigned to the n–σ* transition from P atoms and the C=C π–π* transition in the aromatic ring, respectively.^52^ As discussed above, the rGO spectrum has a strong absorption peak centred at 270 nm (Fig. 2(a)). When combined (*i.e*. PPh_3_–rGO), the features merge to form a shoulder at 230 nm and a narrower peak at 270 nm. The position shift of the PPh_3_ related peaks could be due to the π–π interaction between the ligand and rGO.^52^ The spectrum of Au_101_NC–rGO shows similar absorption bands to PPh_3_–rGO, although the peak at 270 nm is more intense but less distinct. Again, this suggests the formation of non-covalent interactions between the PPh_3_ ligands and rGO in the Au_101_NC–rGO nanocomposite. The lack of an LSPR peak near 520 nm in Au_101_NC–rGO confirms that there is no agglomeration of AuNCs deposited onto rGO made under low pH conditions, as seen in the HAADF-STEM images. Stability of the clusters was tested by measuring the UV-vis absorbance after 1 month (stored in dark, −10 °C) and no LSPR was observed (Fig. S10†), in comparison a sample deliberately agglomerated showed a clear LSPR at 550 nm.

The TGA of Au_101_NC–rGO nanocomposite (in air), shown in Fig. 8(b), was conducted to determine the mass loading of Au from the residual weight. The residual mass was 4.7 w% which we assign to the mass of Au in the sample. The mass loading is similar to the desired loading (5 wt%) and the loading determined from ICP-MS (4.9 wt%), Table 1.

TGA traces for rGO and the Au_101_NC–rGO nanocomposite, obtained under N_2_ flow, are presented in Fig. 8(c). Both samples display a rapid, initial mass loss up to 150 °C due to solvent adsorbed between the rGO sheets, which complicates comparison. Therefore, the % weight loss is set to 100% at this temperature. Both samples exhibit weight loss with two slopes (after solvent loss). The first weight loss occurs due to removal of PPh_3_ ligands on Au_101_NC–rGO (Fig. S4†), and labile functional groups on the rGO. The second weight loss occurs due to the loss of other functional groups from the rGO structure. At 900 °C, the overall weight loss for rGO is 58% (*i.e*. 42% remaining) and for the Au_101_NC–rGO nanocomposite is 29 ± 5% (*i.e*. 71 ± 5% remaining). The extra mass remaining in the Au_101_NC–rGO exceeds the mass of Au in the sample (5%). Therefore, it appears that the presence of Au_101_NC prevents the loss of some rGO functional groups. This again supports our assumption of a non-covalent interaction between the phenyl groups of the PPh_3_ and the rGO support to stabilise the composite.

The combination of information from the characterisation techniques applied give insight on the interaction between Au_101_NC and rGO. Greater than 99% of the pre-synthesised AuNCs were incorporated into the rGO composite (ICPMS) at a relatively high loading of 5 wt% Au. The deposition proceeded quickly under mild deposition conditions which indicates that there is a high affinity between rGO and the Au_101_NC. XPS and ATR-FTIR show that the PPh_3_ ligands remain within the composite after attachment and are therefore most likely responsible for the interaction. With each Au_101_NC containing *ca*. 17 PPh_3_ ligands remaining (based on the ICP-MS estimate) there are 51 phenyl groups decorating each gold cluster. Therefore, π–π stacking and hydrophobic non-covalent interactions between ligands and graphitic structures within rGO are expected to be driving the formation of the nanocomposite. There is a large body of literature also supporting this interaction between aryl ligands such as PPh_3_ and sp^2^ hybridised carbon such as rGO.^53,54^

We anticipate other rGO reduction methods (thermal reduction, chemical, electrochemical) may also be amenable to forming composites with PPh_3_ ligated metal clusters although our findings suggest some guidelines. Any method which uses a high pH or may impart basic functional groups should be avoided as we have observed this will lead to agglomeration of the clusters (Fig. 4). Reduction process should be chosen such that the rGO is highly soluble (to aid in dispersion and reduce stacking) in a good solvent for the cluster and any further processing step (*e.g.* high boiling point and high volatility solvents should be avoided due to difficult processing). Finally, rGO flake size (width and stacking), and degree of reduction should be optimised for any final application in which conductivity, transparency, and dispersion are expected to have an effect.

The simple and mild strategy presented here demonstrates an excellent compatibility between size-specific triphenylphosphine protected gold clusters and rGO which can prevent aggregation of the metal clusters. We expect that this procedure can be applied to any PPh_3_ ligated cluster such as Au_11_(PPh_3_)_8_Cl_3_,^55^ Au_9_(PPh_3_)_8_(NO_3_)_3_,^56^ and Au_8_(PPh_3_)_8_(NO_3_)_2_ (ref. ^19^) as well as triphenylphosphine stabilised clusters of other metals on rGO in order to form agglomeration-resistant cluster–rGO composites, which could have diverse applications.

## 4 Conclusion

In summary, a method was developed for the deposition of the triphenylphosphine-protected Au_101_NCs onto rGO. The results from a wide range of characterization techniques verify the agglomeration-free loading of Au_101_NC with narrow particle size distribution (1.4 ± 0.4 nm) onto the rGO sheets. This has been achieved by a simple modification to the hydrothermal reduction of GO to use aqueous medium at low pH. We hypothesise that the PPh_3_ ligands play an important role in formation composite acting as a non-covalent linking agent resulting in uniform deposition of Au_101_NCs without aggregation.

The proposed methodology provides an easy and convenient avenue towards the preparation of rGO-based nanocomposites with other size-specific phosphine-ligated metal clusters.

## Conflicts of interest

The authors have no conflicts to declare.

## Supplementary Material

NA-003-D0NA00927J-s001
