# Supplementary material for: Au101–rGO nanocomposite: immobilization of phosphine-protected gold nanoclusters on reduced graphene oxide without aggregation
Source: Nanoscale Adv. 2021 Jan 7;3(5):1422–30. doi: 10.1039/d0na00927j (PMC9417812; doi:10.1039/d0na00927j)
Supplement: NA-003-D0NA00927J-s001 [file NA-003-D0NA00927J-s001.pdf]

Electronic Supplementary Material (ESI) for Nanoscale Advances.
This journal is © The Royal Society of Chemistry 2021

# Supporting Information:

# $Au_{101}$–rGO nanocomposite: immobilization of phosphine-protected gold nanoclusters on reduced graphene oxide without aggregation

Hanieh Mousavi[1], Yanting Yin[2], Liam Howard-Fabretto[2], Shailendra Kumar Sharma[3], Vladimir Golovko[3], Gunther G. Andersson[2], Cameron J. Shearer[1*], Gregory F. Metha[1*]

[1] Department of Chemistry, University of Adelaide, Adelaide SA 5005, Australia

[2] Flinders Centre for NanoScale Science and Technology, Flinders University, Adelaide SA 5001, Australia

[3] The MacDiarmid Institute for Advanced Materials and Nanotechnology, School of Physical and Chemical Sciences, University of Canterbury, Christchurch 8140, New Zealand

*Corresponding author: cameron.shearer@adelaide.edu.au, greg.metha@adelaide.edu.au

This supporting information document contains a detailed description about each of the applied characterisation techniques, and also presents the following additional figures and tables:

**Figure S1:** (a) UV-Vis, (b) ATR-FTIR, and (c) TEM image of rGO prepared at pH~11 (top) and pH~2 (bottom).

**Figure S2:** (a) XPS Survey (b) XPS C1s spectrum and (c) Raman spectra, of (i) GO, (ii) rGO (prepared at pH~2), and (iii) rGO (prepared at pH~11).

**Figure S3:** ATR-FTIR spectra of solid $PPh_3$ and $Au_{101}$NC

**Figure S4:** TGA curves of solid $PPh_3$ and $Au_{101}$NC in $N_2$

**Figure S5:** Size distribution of $Au_{101}$NC-rGO (prepared at pH~11) from HAADF-STEM measurements

**Figure S6:** HAADF and elemental mapping of Au and P

**Figure S7:** SEM image of rGO

**Figure S8:** Position of the two Au $4f_{7/2}$ peaks observed in the XPS spectra of $Au_{101}$NC and $Au_{101}$NC-rGO before and after heating to 200 °C

**Figure S9:** XPS C 1s spectrum of (ii) $Au_{101}$NC and (iii) $Au_{101}$NC-rGO and (iii) rGO.

**Table S1:** Fraction of functional groups from XPS presents in (i) rGO, (ii) $Au_{101}$NC and (iii) $Au_{101}$NC-rGO

**Table S2:** XPS peak positions following deconvolution of C 1s, O 1s, Au 4f and P 2p in (i) rGO, (ii) $Au_{101}$NC and (iii) $Au_{101}$NC-rGO

**Figure S10:** UV-vis absorption spectra before and after one month storage at -10 °C and agglomerated $Au_{101}$NC-rGO.

## Characterisation techniques

**Ultraviolet–visible absorption spectroscopy (UV-Vis):** A UV-Vis spectrometer (Cary 5000) was employed in the range of 200-800 nm. All experiments were completed in quartz cells dispersed in solvent (methanol for all samples except GO which was dispersed in water).

**Attenuated total reflectance Fourier transform infrared spectroscopy (ATR-FTIR):** ATR-FTIR spectra (PerkinElmer Spectrum100) were recorded in the range of 500-4000 $cm^{-1}$. For sample preparation, the samples were washed twice with methanol and centrifuged for each wash, followed by drying at room temperature in the dark before analysis. The dried powder was pressed onto the ATR crystal for analysis.

**Scanning electron microscopy (SEM):** The surface morphology, agglomeration state and the distribution of $Au_{101}$NC over the rGO sheets were studied under a High-Resolution Field Emission Scanning Electron Microscope equipped with EDX Silicon Drift Detectors (FEI-SEM Quanta 450).

**Raman spectroscopy:** The vibrational properties of GO and rGO was carried out using Raman spectroscopy (LabRAM Evolution, Horiba Jobin Yvon, Japan and Witec Alpha 300RS) with a 532 nm laser for excitation. All spectra were recorded with an integration time of 10 s for 3 accumulations. For sample preparation, the samples were dropped on a clean glass slide and was then allowed to evaporate to make a thin film before analysis.

**Thermogravimetric analysis (TGA):** A TGA (METTLER TOLEDO) under flow of nitrogenous atmosphere and air, with the dried samples heated from room temperature to 900 °C at the rate of 10 C min $^{-1}$ was applied to investigate thermal stability, composition and ligand binding to the surface of $Au_{101}$NC and rGO**.** The samples were washed twice with methanol and centrifuged for each wash followed by drying at room temperature in dark place overnight before analysis (which typically utilised 5-10 mg of sample).

**Transmission electron microscopy (TEM):** The size, morphology and size distribution of the rGO, $Au_{101}$NC and $Au_{101}$NC-rGO nanocomposite were characterised by a FEI Tecnai G2 Spirit TEM operated at 120 keV and a FEI Titan Themis 80-200 scanning transmission electron microscope (STEM) operating at 80 keV. The high-angle annular dark-field scanning TEM (HAADF-STEM) and STEM-EDS elemental maps were acquired with a FEI Titan Themis STEM operating at 80 keV and equipped with a Super-X EDS detector in conjunction with a low-background sample holder to minimise Cu background peaks and maximise x-ray collection efficiency. EDS data was analysed using Velox™ software from Thermo Fisher Scientific. Samples were prepared by dropping freshly prepared dispersions of as-prepared materials in methanol (sonicated for 1 min) onto a 300-mesh copper grid with a lacey carbon support film for HAADF-STEM and mylar grid for TEM analysis. The solvent was then allowed to evaporate before placing the grid into the sample holder.

**X-ray photoelectron spectroscopy (XPS):** To determine elemental composition and degree of agglomeration of the as prepared materials, X-ray photoelectron spectroscopy (XPS) was conducted. The XPS is operated in UHV with a SPECS PHOIBOS-HSA3500 analyzer with a pass energy of 40 eV for survey spectra and 10 eV for high-resolution spectra. A non-monochromatic Mg $K_\alpha$ X-ray line with an excitation energy of 1,253.6 eV was used as the X-ray source for the analysis. The analysis was conducted in a chamber with a base pressure of a few $10^{-10}$ mbar. The angle between

the incident X-rays and the analyser was 54° and the detection angle of the photoelectrons was 90°. Binding energy calibration was completed in a different manner for samples containing rGO and those without rGO. The $Au_{101}$NC sample (i.e. without rGO) displayed a small amount of charging which was compensated by setting the major C 1s peak to 285.0 eV (see Figure S7 and Table S2), which corresponds to the position of adventitious carbon (compensation was -0.46 eV).[1] For all other samples (i.e. with rGO), no charging was observed and the C position was consistent with graphite $sp^2$ peaks positioned at 284.5 ± 0.15 eV, thus no calibration was performed. For sample preparation, the suspensions of rGO, $Au_{101}$NC and $Au_{101}$NC-rGO in methanol were drop cast onto a clean Si (100) wafer and dried immediately before analysis.

**Inductively Coupled Plasma Mass Spectrometry (ICP-MS):** ICP-MS (Agilent 8900x QQQ) was employed to determine the total content of Au and P in $Au_{101}$NC-rGO nanocomposite by measuring the amount of Au and P that had not adsorbed to rGO. $Au_{101}$NC-rGO nanocomposite (0.5 mL) was suspended in methanol and centrifuged to precipitate solid, followed by filtration of supernatant using Whatman 13 mm, 0.1 µm disposable syringe Nylon filter. Then 0.05 mL of filtrate was taken, and the solvent allowed to evaporate. To dissolve the remaining solid, 0.2 mL of fresh *aqua regia (analysis grade reagents of 32% hydrochloric acid and 70% nitric acid)* was added for several minutes, then filled up to 10 mL with water for analysis. Gold and phosphorous single standard solutions in 2% aqua regia with the concentrations of 5, 10, 25, 50, 100 and 200 ppb were used for calibration.

**Image J and MATLAB software:** Image J and MATLAB were employed to measure the size of gold particles (300 particles) and plot histograms, respectively.

## Results and Discussion:

### Comparison of rGO produced at high and low pH

UV-Vis, FTIR and TEM of rGO produced at high pH is shown in Fig S1. Both UV-Vis and FTIR are similar to rGO produced at low pH. The TEM shows a typical sheet rGO morphology, with a slightly less crumpled morphology than was observed under acidic conditions.

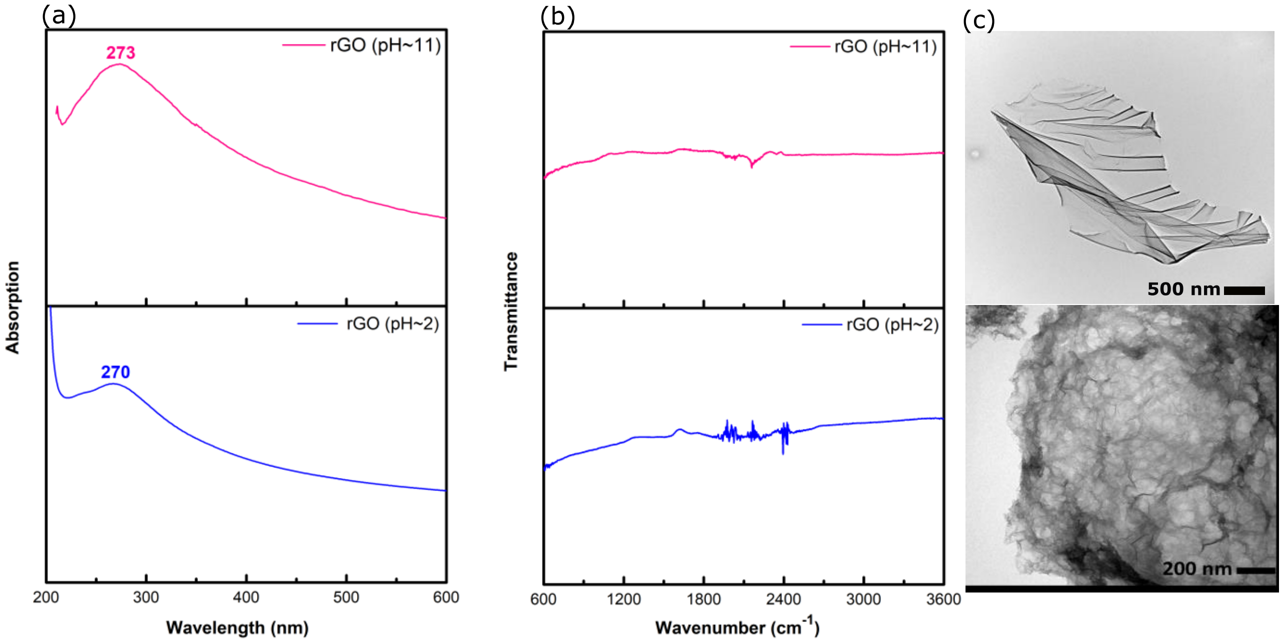


*Figure S1. (a) UV-Vis, (b) ATR-FTIR, and (c) TEM image of rGO prepared at pH~11 (top) and pH~2 (bottom).*

The XPS survey and C 1s region of GO, rGO prepared at pH~2, and pH~11 are presented in Fig. S2(a) and (b), respectively. rGO produced at low pH shows peaks from C, O and a small substrate signal (Ti) at 285, 530 and 459 eV. The rGO prepared at high pH did not show the substrate peak (better sample preparation), but did show a peak from nitrogen (400 eV), likely from the ammonia added during reduction. The spectrum of each are fit to five main peaks: 284.8 C=C-C ($sp^2$), 285.7 (C–C ($sp^3$)), 286.8 (C–O), 287.5 (C-O-C/C=O), and 289.0 (O-C=O) eV. As expected the GO has a high proportion of oxidised carbon (C-O, C=O) which significantly reduce upon reduction.

Comparing the relative area of C=C-C $sp^2$ and C–O bonds confirm that the ratio of $I_{C–C}/I_{C–O}$ from 1 in GO to 4.3 and 3.6 in rGO (pH~2), and rGO (pH~11), respectively. The fraction of each functional group present in GO, rGO (pH~2), and rGO (pH~11) and the analysis from deconvolution of C 1s and O 1s spectra are shown in Table S1 and Table S2. In summary, the reduction methods have reduced the GO to a similar extent and the reduction is not complete with a relatively high amount of oxygen functional groups remaining (when compared to rGO via hydrazine or high temperature reduction).[2] Due to the stability of the gold clusters in methanol we aimed to produce rGO which was still highly soluble in methanol. Furthermore, some remaining functional groups reduce stacking of rGO layers in solution to maintain a high surface area.

The structural and chemical composition changes of GO and rGO were evaluated by Raman. The Raman spectra of GO and rGO prepared at pH~2, and pH~11 is shown in Fig. S2(b). The spectra show the G (~1590 cm$^{-1}$) and D (~1350 cm$^{-1}$) band related to vibrational of C-C=C ($sp^2$) and the presence of the structural defects ($sp^3$) of the graphitic domains, respectively.[3] The calculated $I_D/I_G$ ratio for GO and rGO prepared at pH~2, and pH~11 increased to 0.93, 1.05 , and 1.07 respectively.

The combined analysis of the graphene reduced at different conditions yields only small differences in material with the major difference being the presence of nitrogen when reduced in

basic conditions, which may in the form of basic ammonia groups. Although altering the reduction method further was not investigated in this work, it is expected to play a significant role in application of the final devices and reduction will likely required to be optimised for each purpose.

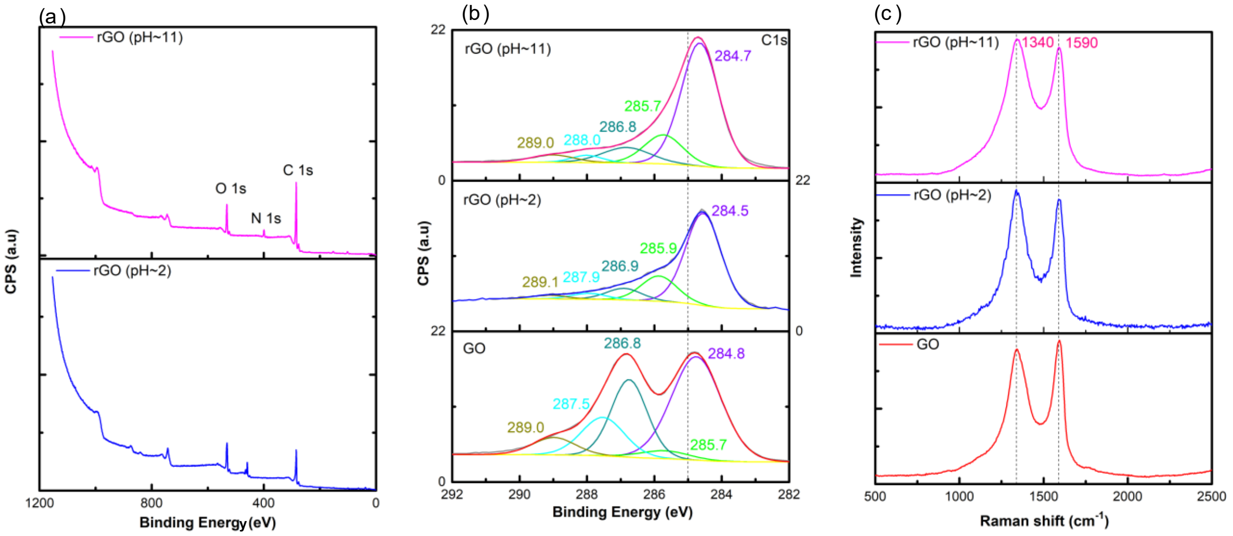


*Figure S2. (a) XPS Survey (b) XPS C1s spectrum and (c) Raman spectra, of (i) GO, (ii) rGO (prepared at pH~2), and (iii) rGO (prepared at pH~11).*

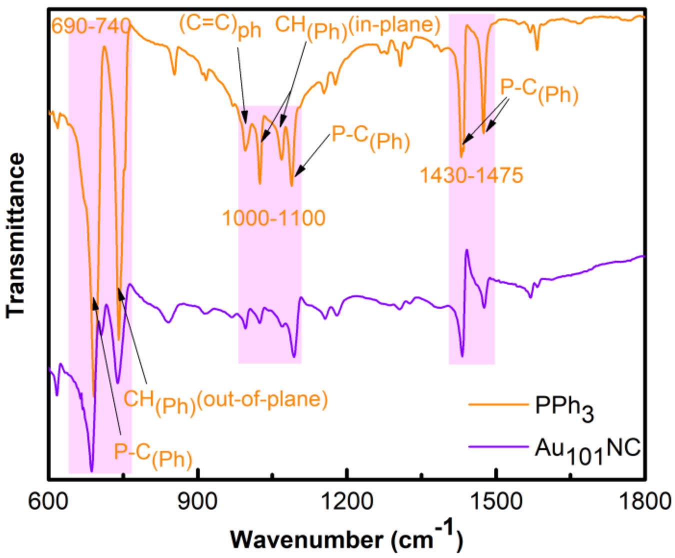


*Figure S3. ATR-FTIR spectra of solid $PPh_3$ and $Au_{101}NC$.*

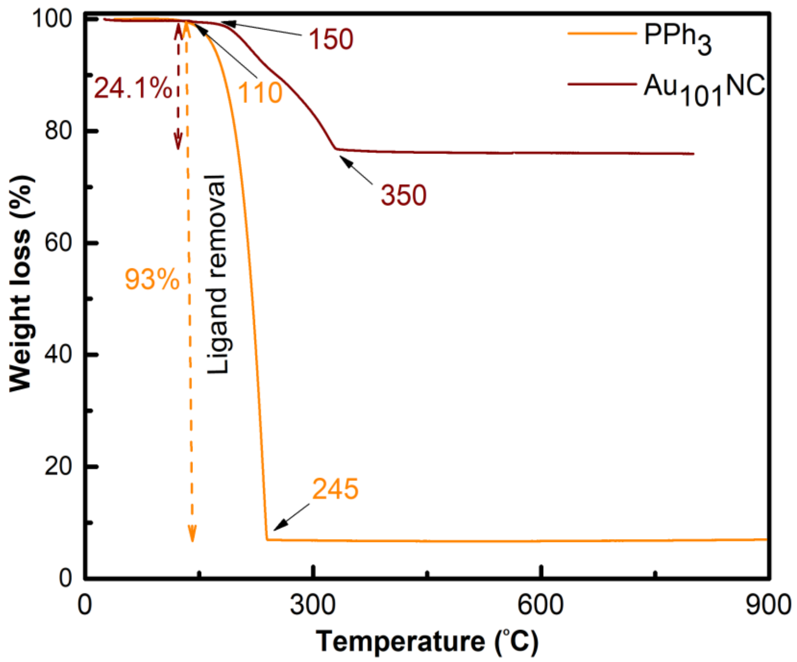


Figure S4. TGA curves of solid $PPh_3$ and $Au_{101}$NC in $N_2$.

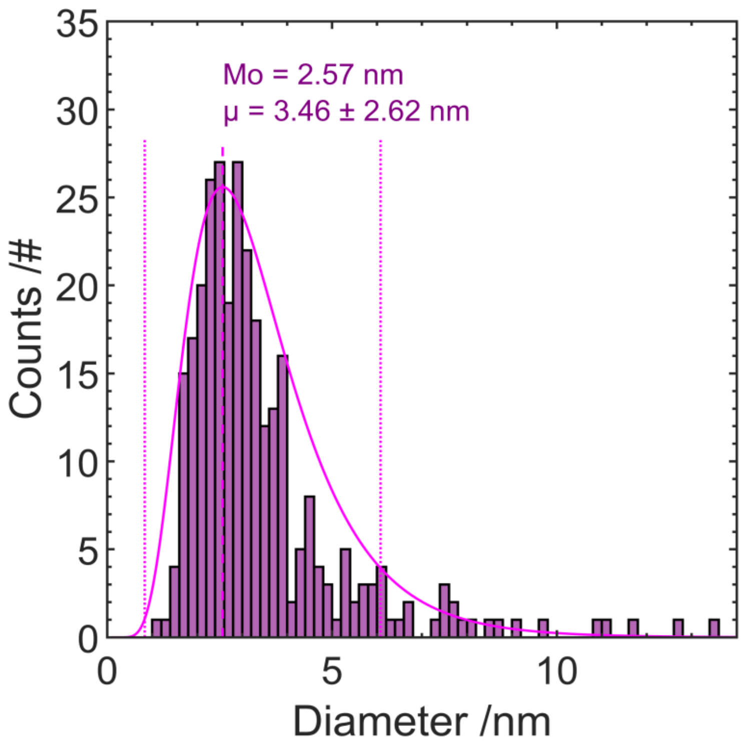


*Figure S5. Size distribution of $Au_{101}$NC-rGO (prepared at pH~11) from TEM measurements*

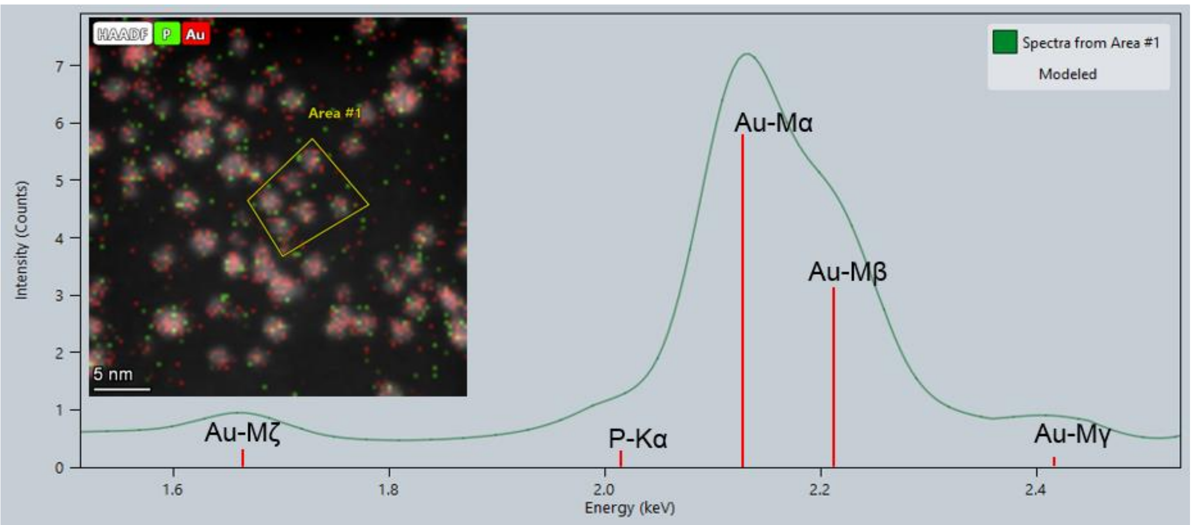


*Figure S6. Modelled to fit integrated spectrum for $Au_{101}$-rGO sample from HAADF-STEM EDS data. Insert shows the HAADF image overlaid with a map of wt% Au and P*

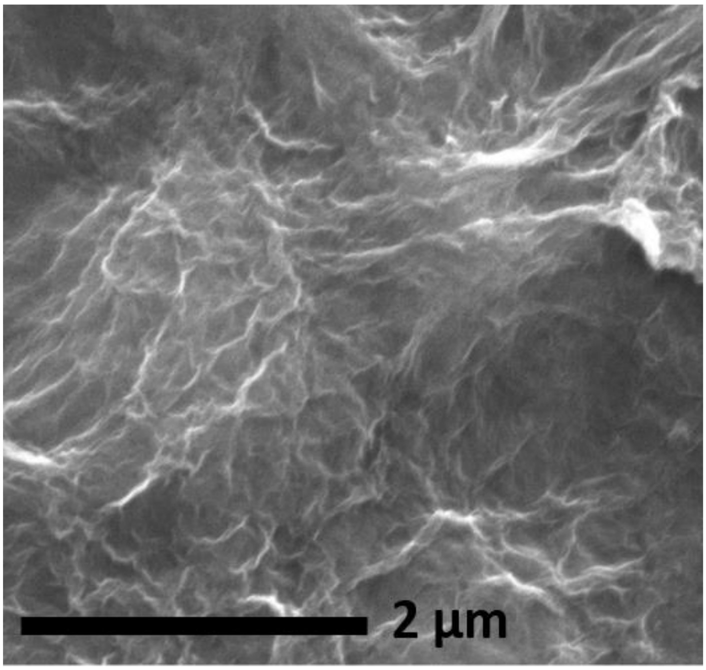


*Figure S7. SEM image of rGO*

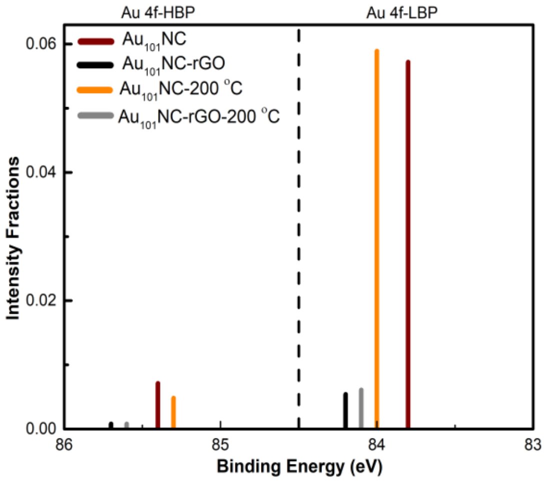


*Figure S8. Position of the two Au 4f$_{7/2}$ peaks observed in the XPS spectra of Au$_{101}$NC and Au$_{101}$NC-rGO before and after heating to 200 °C.*

To investigate the strength of the binding between the Au$_{101}$NC and rGO as monitored by XPS, the Au$_{101}$NC, and Au$_{101}$NC-rGO nanocomposite were heated *in situ* to 200 °C in an attempt to induce agglomeration. After heating phosphorus was no longer observed which indicates the clusters were delegated. The position of the two Au 4f$_{7/2}$ peaks are summarised in the bar chart presented in Fig. S6. For Au$_{101}$NC, the 4f$_{7/2}$ LBP peak shifts slightly to higher energy, whereas the HBP shifts lower energy upon heating. For the Au$_{101}$NC-rGO nanocomposite, both the 4f$_{7/2}$ LBP and HBP peaks shift slightly to higher energy. This suggests that the smaller clusters have agglomerated into larger sized clusters but not quite into bulk-like gold particles.[4] This indicates the important role of rGO yielding the strong interaction with AuNCs, resulting in increased stability and agglomeration resistance of the Au$_{101}$NC-rGO nanocomposite upon heating. [5, 6]

*Table S1. Fraction of functional groups from XPS present in (i) rGO, (ii) $Au_{101}$NC and (iii) $Au_{101}$NC-rGO. Standard error in values is 10%.*

| | | C 1s% | | | | | | O 1s% | | | Au 4f% | | P 2p% | | | |
|---|---|---|---|---|---|---|---|---|---|---|---|---|---|---|---|---|
| | | C $sp^2$ (C=C-C) | C# | C $sp^3$ (C-C) | C-OH | C-O-C/ C=O | HO-C=O | O 1s-1 | O 1s-2 | O 1s-3 | Au 4f - LBP | Au 4f-HBP | P $2p_{3/2}$ LBP | P $2p_{1/2}$ LBP | P $2p_{3/2}$ HBP | P $2p_{1/2}$ HBP |
| | **GO** | **48.0** | **-** | **3.6** | **25.9** | **15.5** | **7.0** | **34.1** | **24.1** | **41.8** | - | - | - | - | - | - |
| | **rGO (pH~11)** | **65.7** | **-** | **15.9** | **10.7** | **3.2** | **4.6** | **40.9** | **21.1** | **38.0** | - | - | - | - | - | - |
| **i** | **rGO (pH~2)** | 66.5 | | 18.2 | 8.3 | 4.3 | 2.7 | 54.6 | 28.2 | 17.3 | - | - | - | - | - | - |
| **ii** | **$Au_{101}$NC** | - | 93.8 | | 6.2 | - | - | 36.6 | 52.1 | 11.3 | 88.9 | 11.1 | 58.5 | 29.3 | 8.1 | 4.1 |
| **iii** | **$Au_{101}$NC-rGO** | 74.2 | | 12.1 | 8.7 | 3.2 | 1.8 | 25.4 | 36.8 | 37.8 | 87.1 | 12.9 | 66.7 | 33.3 | - | - |

# resulting from a mixture of adventitious carbon ($sp^3$) and the C from the triphenylphosphine ligands ($sp^2$).

*Table S2. Deconvolution of C 1s, O 1s, Au 4f and P 2p in (i) rGO, (ii) $Au_{101}$NC and (iii) $Au_{101}$NC-rGO*

| | | Au4f | | | | P2p | | | |
|---|---|---|---|---|---|---|---|---|---|
| | | Au $4f_{7/2}$ LBP | Au $4f_{5/2}$ LBP | Au $4f_{7/2}$ HBP | Au $4f_{5/2}$ HBP | P $2p_{3/2}$ LBP | P $2p_{1/2}$ LBP | P $2p_{3/2}$ HBP | P $2p_{1/2}$ HBP |
| **i** | **rGO** | - | - | - | - | - | - | - | - |
| **ii** | **$Au_{101}$NC** | 83.8 | 87.5 | 85.4 | 89.1 | 131.3 | 132.1 | 133.3 | 134.1 |
| **iii** | **$Au_{101}$NC-rGO** | 84.2 | 87.9 | 85.7 | 89.3 | 131.2 | 132.0 | - | - |

| | | C1s | | | | | | O1s | | |
|---|---|---|---|---|---|---|---|---|---|---|
| | | C $sp^2$ (C=C-C) | C# | C $sp^3$ (C-C) | C-OH | C-O-C/ C=O | O-C=O | O 1s-1 | O 1s-2 | O 1s-3 |
| | **GO** | **284.8** | **-** | **285.7** | **286.8** | **287.5** | **289.0** | **531.7** | **532.6** | **533.3** |
| | **rGO (pH~11)** | **284.7** | **-** | **285.7** | **286.8** | **288.0** | **289.0** | **531.5** | **532.1** | **533.0** |
| **i** | **rGO (pH~2)** | 284.5 | - | 285.9 | 286.9 | 287.9 | 289.1 | 530.9 | 532.4 | 533.7 |
| **ii** | **$Au_{101}$NC** | - | 285.0* | | 286.2 | - | - | 531.5 | 532.2 | 533.8 |
| **iii** | **$Au_{101}$NC-rGO** | 284.5 | | 285.7 | 286.4 | 287.6 | 288.8 | 531.0 | 532.3 | 533.5 |

# resulting from a mixture of adventitious carbon ($sp^3$) and the C from the triphenylphosphine ligands ($sp^2$).

* Calibrated value (shift was -0.46 eV)

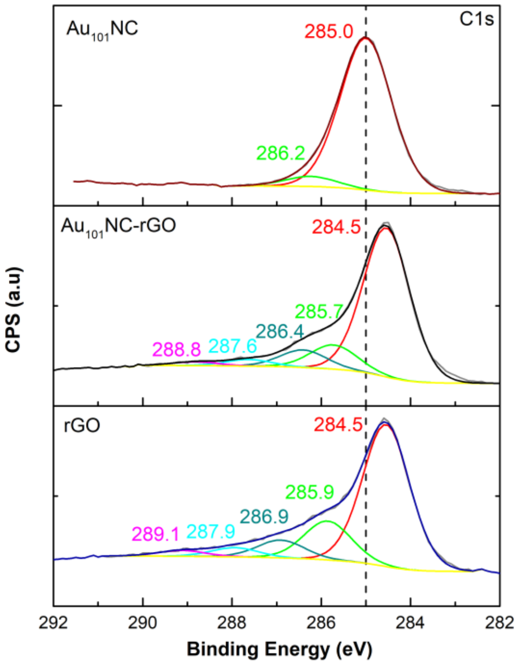


**Figure S9:** XPS C 1s spectrum of (ii) $Au_{101}$NC and (iii) $Au_{101}$NC-rGO and (iii) rGO.

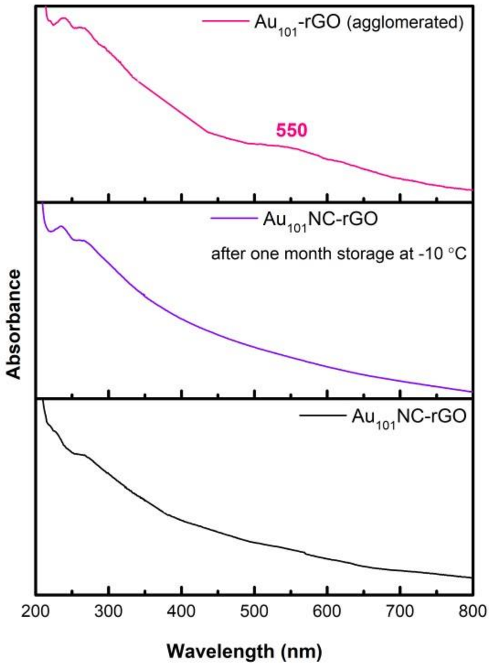


**Figure S10:** UV-vis absorption spectra before and after one month storage at -10 °C and agglomerated $Au_{101}$NC-rGO.

## Supporting References

1. D. P. Anderson, J. F. Alvino, A. Gentleman, H. A. Qahtani, L. Thomsen, M. I. J. Polson, G. F. Metha, V. B. Golovko and G. G. Andersson, *Physical Chemistry Chemical Physics*, 2013, **15**, 3917-3929.

2. H. A. Becerril, J. Mao, Z. Liu, R. M. Stoltenberg, Z. Bao and Y. Chen, *ACS Nano*, 2008, **2**, 463-470.

3. R. Muzyka, S. Drewniak, T. Pustelny, M. Chrubasik and G. Gryglewicz, *Materials*, 2018, **11**, 1050.

4. H. S. Al Qahtani, G. F. Metha, R. B. Walsh, V. B. Golovko, G. G. Andersson and T. Nakayama, *The Journal of Physical Chemistry C*, 2017, **121**, 10781-10789.

5. C. Wang, N. Li, Q. Wang and Z. Tang, *Nanoscale research letters*, 2016, **11**, 336.

6. Q. Wang, L. Wang, Z. Tang, F. Wang, W. Yan, H. Yang, W. Zhou, L. Li, X. Kang and S. Chen, *Nanoscale*, 2016, **8**, 6629-6635.
